# Transcriptome Analysis of Synaptoneurosomes Identifies Neuroplasticity Genes Overexpressed in Incipient Alzheimer's Disease

**DOI:** 10.1371/journal.pone.0004936

**Published:** 2009-03-19

**Authors:** Celia Williams, Ruty Mehrian Shai, Yongchun Wu, Ya-Hsuan Hsu, Traci Sitzer, Bryan Spann, Carol McCleary, Yi Mo, Carol A. Miller

**Affiliations:** 1 Department of Pathology, Keck School of Medicine University of Southern California, Los Angeles, California, United States of America; 2 Department of Neurology, Keck School of Medicine University of Southern California, Los Angeles, California, United States of America; 3 Program in Neuroscience, Keck School of Medicine University of Southern California, Los Angeles, California, United States of America; 4 Norris Medical Library, Keck School of Medicine University of Southern California, Los Angeles, California, United States of America; 5 Biochemistry and Molecular Biology, Institute for Genetic Medicine, Keck School of Medicine University of Southern California, Los Angeles, California, United States of America; University of Nebraska, United States of America

## Abstract

In Alzheimer's disease (AD), early deficits in learning and memory are a consequence of synaptic modification induced by toxic beta-amyloid oligomers (oAβ). To identify immediate molecular targets downstream of oAβ binding, we prepared synaptoneurosomes from prefrontal cortex of control and incipient AD (IAD) patients, and isolated mRNAs for comparison of gene expression. This novel approach concentrates synaptic mRNA, thereby increasing the ratio of synaptic to somal mRNA and allowing discrimination of expression changes in synaptically localized genes. In IAD patients, global measures of cognition declined with increasing levels of dimeric Aβ (dAβ). These patients also showed increased expression of neuroplasticity related genes, many encoding 3′UTR consensus sequences that regulate translation in the synapse. An increase in mRNA encoding the GluR2 subunit of the α-amino-3-hydroxy-5-methyl-4-isoxazole propionic acid receptor (AMPAR) was paralleled by elevated expression of the corresponding protein in IAD. These results imply a functional impact on synaptic transmission as GluR2, if inserted, maintains the receptors in a low conductance state. Some overexpressed genes may induce early deficits in cognition and others compensatory mechanisms, providing targets for intervention to moderate the response to dAβ.

## Introduction

Synaptic dysfunction and loss in Alzheimer's disease (AD) are correlates of cognitive impairment [1], [2] emphasizing the importance of identifying early molecular changes at the synapse. Early in the course of AD, synaptic dysfunction, which is present before the accumulation of histopathological hallmarks of AD, may be reversible [3], [4].

Initially, amyloid plaque pathology in AD was thought to correlate positively with clinical progression of the disease [5] until the toxic effects of soluble beta-amyloid (Aβ) peptides, both Aβ1-40 and Aβ 1-42, were strongly associated with cognitive decline even in the absence of significant tau pathology [6]. oAβ is implicated specifically in targeting the synapse by binding to cell surface receptors [7]–[9], an interaction which provides a molecular basis for reversible memory loss in the Tg2576 transgenic mouse model of AD [10], [11]. Accumulation of dAβ in transgenic mice occurs concurrently with memory impairment, suggesting a causal role [12] although, trimers have also been suggested as the most active toxic species in mouse hippocampal, long term potentiation (LTP) inhibition [13]. In rats, intracerebroventricular injection of oAß induces transient disruption of working memory [14], [15] and organotypic slices incubated with oAβ show loss of dendritic spines and a decrease in excitatory synapses mediated by activity of N-methyl-d-aspartate receptor (NMDARs) through a pathway involving cofilin and calcineurin [16] These conditions favor reduction in LTP and facilitation of long term depression (LTD) and, although LTP and LTD are experimental *in vitro* phenomena, a comparable modification in synaptic plasticity may occur *in vivo*, early in AD. Aß also impacts AMPAR mediated currents [17]. Studies showing inhibition of AMPAR transmission after bath application of Aß [18] and synaptic silencing through a selective reduction in AMPARs in primary cultured neurons overexpressing amyloid precursor protein (APP) [19] infer a crucial role in AD pathogenesis. However, mouse models do not reflect the full pathogenesis of AD and although human genetic, epigenetic and behavioral variability add complexity to such analyses, the ability to use the human central nervous system (CNS) provides a very powerful and corroborative approach for understanding disease progression [20]. Recent reports have pushed the boundaries of experiments using postmortem human CNS tissue in complex diseases, such as schizophrenia, [21] .

Microarray analyses of the human CNS have been used extensively to define changes in gene expression in pathways of learning and memory [22], aging [23], and neurodegenerative diseases [24]. Previous microarray studies compare whole homogenates or even single cells obtained postmortem from control and AD brains [25]–[29] and provide data that is relevant to the disease. Blalock [30] analyzed hippocampal tissue obtained from patients with minimal cognitive impairment (MCI) as well as more severe dementia to target early changes in gene expression. We also focus on early changes in mRNA expression that may occur prior to formation of amyloid plaques and neurofibrillary tangles (NFTs). Our study examines a spectrum of patients ranging from controls to those with incipient AD (IAD), which combines patients with MCI and early AD categorized by neuropsychological changes in global executive and memory functions. As the hippocampus sustains neuropathological changes early in the disease and few patients come to autopsy with only entorhinal cortical change, we analyze postmortem prefrontal cortex (Brodmann's areas 9 and 10) with absent or minimal Aβ or tau fibrilization. In AD, prefrontal cortex is the site of early neocortical changes [31], and also provides sufficient quantities of tissue for microarray and biochemical analysis. Also, other limbic system sites, such as anterior cingulate gyrus, cannot be readily neuropsychologically evaluated.

Plasticity, memory encoding and consolidation are dependant on local protein synthesis, initiated and regulated on site within the synapse [32], [33]. Thus perturbation by Aβ of receptor binding and signaling at the synapses may disrupt local translation and thereby initiate the cascade of molecular events which negatively impact cognition and initiate neurodegeneration. The relevance to AD of 3′UTR regulatory sequences and local protein synthesis at the synapse are largely unexplored. In primary rat neuronal cultures, axons, dendrites and their synaptic terminals contain only about 3.9% of the cellular total RNA [34]. Thus, functionally significant changes in synaptic mRNAs during the course of AD may be masked by more abundant species in microarray analyses using total cellular mRNA isolated from neuronal somata, glia and other cells. To elucidate the effects of oAβ on synaptic mRNA, we used synaptoneurosomes prepared from the prefrontal cortex to enrich synaptic mRNAs for microarray analyses.

The sensitivity of our approach reveals early expression change in neuroplasticity genes, including those regulated and translated at the synapse. The GluR2 subunit of AMPAR shows elevated gene and protein expression in IAD concomitant with elevating levels of dAβ. Our results may suggest a spectrum of therapeutic targets during dementia progression in AD.

## Results

### Cognitive function and neuropathology

Global cognition and function were evaluated by the Mini-Mental Status Examination (MMSE) and Clinical Dementia Rating Scale (CDR) testing (see Supplemental Methods S1 and data Table S1). Our subjects were categorized primarily on MMSE data into two groups: controls (MMSE 30-25) and “Incipient AD” (MMSE 21-26, with MCI 24-26 and mild AD 21-23). Some subjects in the control group had amyloid plaques and/or NFTs. We reasoned that patients with pathology yet good cognitive function may yield insights into protective or compensatory mechanisms. After careful review of neuropsychological and neuropathological data, two borderline cases both with MMSE 25 (1 Control and 1 IAD) were assigned based not only on MMSE, but also NFT, amyloid plaque and Braak stage data (not shown). A clear distinction is not always possible within the transitional border between control and MCI. We considered MMSE and CDR as well as the neuropathology, especially, NFTs. For example in Table S1, the last control shows an MMSE of 25 and CDR 0.5, but the prefrontal cortex has no pathology. The hippocampal section showed minimal NFTs (not shown) and an overall Braak score of III. Mild depression was noted by neuropsychologists at the last patient interview which may have lowered her test scores. The first patient in the IAD set has an MMSE of 25, CDR1 and again no prefrontal cortical neuropathologic changes. However, the hippocampus has severe NFT's (not shown) yet a Braak score of III and was placed in the IAD group. Overall, the variability of MMSE, and the indistinct Braak values complicate diagnoses at transitional points.

There were no significant group differences in executive function measures; the composite or Digit Span backward (p>0.05), (Table S1). There was a trend for a decline in recall with controls recalling more words after a delay than IAD patients (p<0.08). These results are more indicative of neuropathological changes in the hippocampus as reflected in most Braak scores. Controls showed no significant change in working memory, the ability to temporarily store and manipulate information (p<0.13). Statistical interpretation must be made with caution given the small sample size. No significant group differences were found with measures of memory retention.

### Enrichment and functional stability of synaptic mRNAs

**Figure 1:**
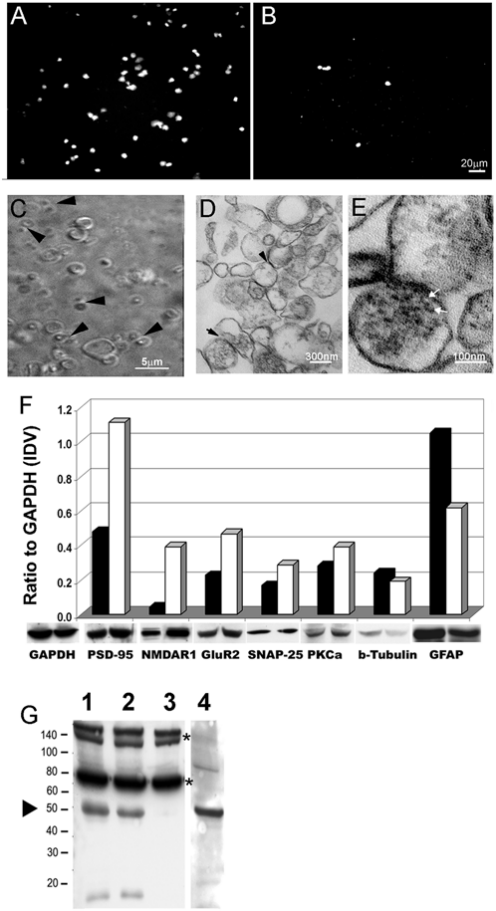
Enrichment and stability of synaptoneurosomes. Microscopy: A) Nuclear contamination of the homogenate (2 µl smears) (DAPI fluorescence) decreases after sequential passage through mesh screens with B) the post- 10 µm screen synaptoneurosome pellet (100 µl volume) still retaining a few nuclei (Bar = 20 µm). C) Intact synaptoneurosomes are detected by phase contrast in smears of pelleted post-10 µm screen synaptoneurosome as typical “snowman” pre- and postsynaptic profiles (arrowheads). Larger, empty, membranous structures are also observed. (Bar = 5 µm). D) Electron microscopy reveal “snowman” profiles with apparent postsynaptic densities (arrowheads); (Bar = 300 nm). E) At higher magnification, presynaptic terminals containing synaptic vesicles, 20 nm in size (white arrows) are indicated (Bar = 100 nm). Immunoblots: F) Homogenates (black bars) and synaptoneurosomes (white bars) were compared for stability and enrichment of synaptic proteins and contamination with other cellular debris. Densitometric comparison on immunoblots of postsynaptic proteins PSD-95, NMDAR1 and GluR2 in synaptoneurosomes shows more than a two-fold enrichment compared to homogenates, an increase in presynaptic protein SNAP-25 but a 40% decrease in glial protein GFAP. Cytoplasmic proteins β-tubulin and PKCα show little change. The ratio of each protein to GAPDH is given in arbitrary units based on the integrated density value which is the sum of all pixel values after background correction (IDV). G) In synaptoneurosomes, de novo synthesis of several proteins is observed as newly translated, biotinylated proteins detected with streptavidin (lane 1), and one at 50 kD co-migrates with a band detected with antibody to αCAMKII (lane 4,arrowhead). Actinomycin D, a transcription inhibitor, does not reduce the protein band profile (lane 2). Protein synthesis is inhibited by the translation inhibitor, anisomycin (lane 3), although endogenously biotinylated proteins present in synaptoneurosomes are still seen at ∼140 and 75 kD (asterisks).

To augment studies comparing homogenates of control to AD brains, we used synaptoneurosome preparations [35] to enrich synaptic mRNAs. Synaptoneurosomes were prepared from the prefrontal cortices of control and IAD patients by a simple, rapid, but gentle method using sequential mesh screens. Preparations were analyzed for residual nuclei by applying 4′-6-Diamidino-2-phenylindole (DAPI)-containing mounting media to smears of each pellet. The percentage of nuclei in the synaptoneurosome pellet was reduced to 0.4% of those in the homogenate (Figure 1A, 1B) and are, therefore, a minor source for contaminating mRNAs. Most nuclei were observed densely packed in the first pellet (P1, not shown).

Morphologic integrity of synaptoneurosomes was confirmed by phase contrast microscopy. We observed pre-and postsynaptic “snowman” profiles (0.3–0.7 µm) in the size range, expected for synaptoneurosomes (Figure 1C). Ultrastructurally, these profiles consist of intact pre- and postsynaptic terminals with typical synaptic vesicles in the presynaptic terminal and a well-preserved postsynaptic density (Figure 1D, E) comparable to those isolated from rat brain [35]. These observations suggest that sufficient synaptoneurosomes remain morphologically intact for extraction of synaptic mRNAs. Myelin profiles and empty membranous structures were also noted.

To verify preservation and enrichment of synaptic proteins, we probed immunoblots of homogenates and synaptoneurosome pellets from normal controls with antibodies to synaptic proteins and other potential contaminating species. Immunoreactive proteins were quantified by densitometry and normalized to glyceraldehyde-3-phosphate dehydrogenase (GAPDH) detected in each sample lane. There is an increase in post-synaptic density protein 95 (PSD-95) (×2.3), NMDAR1 (×8.0), GluR2 (×2.1) and synaptosomal-associated protein, 25 kDa (SNAP25) (×1.7) in synaptoneurosomes compared to homogenates. Using PSD-95 for comparison, our enrichment of synaptic proteins is similar to that shown by others [36], [37] and with mouse synaptoneurosomes prepared in our laboratory (results not shown) . Protein kinase Cα (PKCα), a cytoplasmic proteins which is active in synapses, is slightly more abundant in synaptoneurosomes (×1.4) but β-tubulin (×0.8) is not. A decrease (×0.4) in glial fibrillary protein (GFAP), indicates reduction of astrocytes comparable to that found by others [38] (Figure 1F).

The functionality of mRNAs was assessed by an *in vitro* translation assay. To detect translation products, we used Transcend™ tRNA which is an ε-labeled biotinylated lysine-tRNA complex with a detection sensitivity of 0.5–5 ng of protein. Initially, we used pooled mRNA isolated from the frontal cortices of either control or IAD patients and incubated with rabbit reticulocyte lysate (RRLs) and Transcend™ tRNA. Newly synthesized, biotinylated proteins were detected on immunoblots (Figure S1A). Next, to determine if the postsynaptic translation apparatus is functional, we incubated the synaptoneurosomes with Transcend biotinylated tRNA, but without RRLs. We observed several bands representing newly translated biotinylated protein detected with streptavidin (Figure 1G,). One lane, containing only the basal reaction, was probed with antibody to the α-subunit of Ca^++^ calmodulin-dependant protein kinase II (αCAMKII) and revealed a band with identical migration to a newly synthesized protein at 50 kD. Although this is not definitive proof of the identity of the 50 kD band, synthesis of αCAMKII has been demonstrated previously in synaptoneurosomes isolated from rat brain [39]. Incubation with actinomycin, a transcription inhibitor, did not change the band profiles. However in samples incubated with the translation inhibitor anisomycin newly synthesized bands were not detected. Endogenously biotinylated bands at ∼140 and ∼75 kD were found in all lanes of all immunoblots of synaptoneurosomes detected with streptavidin, and serve as loading controls [40]. To confirm the reproducibility of the translation assay we tested synaptoneurosomes from three controls and two IAD patients. Basal translation profiles (Figure S1B) show bands, other than endogenously biotinylated bands, at a range of molecular weights similar to results from mouse synaptoneurosomes [36], [39]. An increase in the amount of protein loaded and variations in Western blotting and detection are likely factors influencing the number of bands detected. Our results indicate that synaptoneurosomes isolated from human postmortem brain tissue contain functional mRNA and the protein translation components necessary for *de novo* synthesis of proteins from locally positioned mRNA.

### Oligomeric Aβ Expression

**Figure 2:**
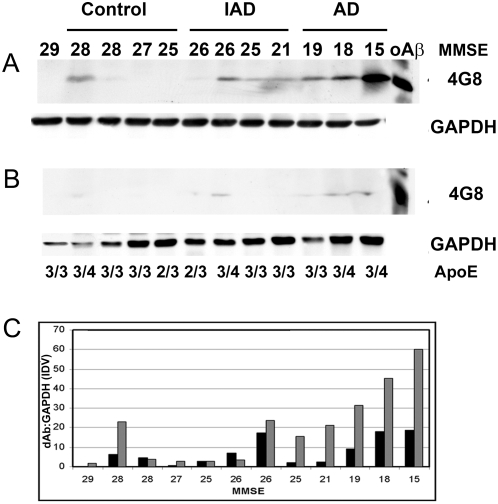
Oligomeric Aβ increases with dementia progression. Oligomeric Aβ dimer (dAβ) is detected with antibody 4G8 on immunoblots of A) whole homogenate or B) synaptoneurosomes prepared from patients with a range of MMSE scores (29-15). A band observed at <10 kD with a migration identical to dAβ, is detectable in IAD patients with MMSE scores of about 26 or less. In C) densitometry reveals that, when normalized to GAPDH, dAβ levels are inversely related to MMSE although patients with an *ApoE4* allele (including one control) exhibit higher concentrations of dAβ than patients with comparable MMSE scores but no E4 allele. Homogenate (grey bar) and synaptoneurosome (white bar) samples show increasing dAβ in IAD as the disease progresses. Data are representative of 3 separate experiments. The ratio of dAβ to GAPDH is given in arbitrary units based on the integrated density value which is the sum of all pixel values after background correction (IDV).

To correlate dementia profiles with oAβ expression, Western blots of homogenates from the frontal cortices of control, IAD and moderate to severe AD patients were probed with antibody 4G8, which detects oAβ1-42. A ∼9 kD band consistent with the dAβ was found and increased in intensity with degree of cognitive impairment, as defined by declining MMSE scores (Figure 2A, C). Additional bands of higher molecular weight were identified by MAb 4G8 in homogenates, but their variability of expression was not consistent with disease progression (Figure S2A). For instance, densitometric comparison of dAβ in control compared to IAD and control compared to AD patient groups showed a consistent increase as the disease progressed whereas an apparent tetramer (tAβ) did not increase in IAD and declined in AD (Figure S2B). Because of the small number of samples in each group, and variability within control and IAD groups, the change in dAβ is not significant. To discriminate the dAβ from the C-terminal stub, CT-83, which migrates close to 8 kD we used MAb 6E10 which confirmed the ∼9 kD species as dAβ (Figure S2C). Another predictor of dAβ expression is the *ApoE4* genotype, as dimers were detected in all patients with at least one *ApoE4* allele, including one control. A later evaluation of dAβ in IAD and AD with more patients in the IAD and AD groups, but without the confounding effect of one control with *ApoE4* genotype and increased dAβ, showed the elevation of dAβ levels to be significant (data not shown). dAβ abundance in synaptoneurosome preparations also increased as the MMSE declined (Figure 2B). Comparison of the Aß dimer normalized to GAPDH in homogenates and synaptoneurosomes showed a consistent quantitative increase with the duration of disease (Figure 2C).

### Microarray analysis

The yield of RNA (10 µg) from synaptoneurosomes prepared from 1gram of tissue was sufficient for probe generation. Only samples with well defined ribosomal peaks at 28S and 18S were included in the study. All 15 patients initially chosen for our study yielded mRNA suitable for microarray analysis with one preparation discarded later due to poor hybridization. Bioinformatics analysis revealed moderate degradation of cRNA transcripts towards the 5′ end in most samples. However, varying degrees of degradation create a reduction in transcript length, not a reduction in the amount of transcripts, and agonal state, postmortem interval (PMI) and pH have not been found to correlate significantly with RNA degradation [41]. Stringent protocols cannot be fully imposed on postmortem human tissue and only PMI can be somewhat controlled by efficient coordination of autopsy procedures. Initial RNA transcript quality has some effect on the microarray absolute call and signal strength but later quality control screens identify samples with significant degradation as outliers.

**Figure 3:**
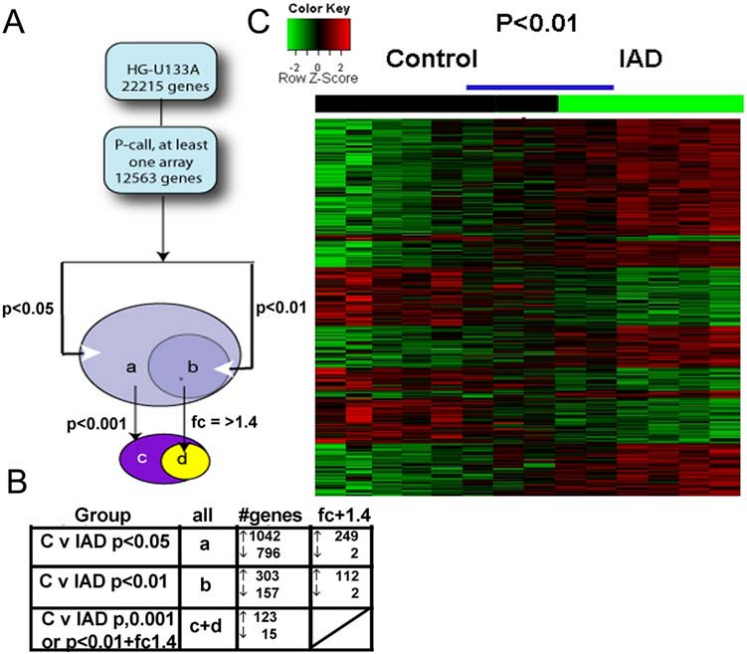
Microarray and Cluster Analysis. In A) Genes were filtered out on the “present” call (P-call) generated by MAS 5.0 leaving 12,536 genes. A 2-way ANOVA test performed on log10-transformed data comparing control with IAD detects the significantly expressed genes. Changes in gene expression in the IAD patients were evaluated at 3 levels of significance (p<0.05, p<0.01, p<0.001) for patients grouped by MMSE values and summarized in the chart below, B). We used the p<0.01 analysis gene list for further study. In C), cluster analysis shows relative mRNA message in controls (black bar) and IAD patients (green bar) with increased expression (green to red) or decreased expression (red to green). Blue line above the heatmap encompasses patients with gene profiles transitional between control and IAD. See Table S2 for genes lists with gene name, symbol and fold change.

The Affymetrix Human Genome HG-U133A chip contains 22,215 transcripts. A 2-way ANOVA test was applied to identify genes with changed expression at three levels of significance p<0.05, p<0.01 and p<0.001or p<0.01 with 1.4+fold change (fc) (Figure 3A). The ratio of genes with increased compared to decreased expression in IAD was 1.3 fold at p<0.05, 1.8 fold at p<0.01 and 8 fold at p<0.001or p<0.01fc 1.4 (Figure 3B). Gene name, gene symbol, functional classification and p-value of all genes are given in supplementary data, (Table S2). We used the p<0.01 analysis for hierarchical clustering which reveals a spectrum of gene expression changes in the heatmap (Figure 3C) with 5 patients (3 controls and 2 IAD) under the blue bar, seemingly on the cusp of the more dramatic changes seen in IAD patients on the far right. The data discussed in this publication are MIAME (Minimum Information About a Microarray Experiment) compliant and have been deposited in the Gene Expression Omnibus (GEO) at the National Center for Biotechnology Information [42], and is accessible through GEO Series accession number GSE12685 (http://www.ncbi.nlm.nih.gov/geo/query/acc.cgi?acc12685).

### Functional analysis of genes overexpressed in IAD

The analytical tools at DAVID Bioinformatic Resources (http://david.abcc.ncifcrf.gov) were used to generate a list of gene ontology categories containing genes with changed expression in IAD. All genes in the p<0.01 list were included in the analysis regardless of fold change. We focused on the category Biological Processes and identified 11 significantly overrepresented themes (EASE score <0.06) constituting four categories (Table 1), including synaptic transmission (p<0.013), neuronal cell-recognition (p<0.005) and vesicle transport (p<0.007), all essential for synaptic function and plasticity. One other category, glucose catabolism, may be related to more general metabolic changes in IAD [43]. Chromatin remodeling (p<0.06) and regulation of cell-cycle (p<0.015) categories contain downregulated transcripts.

**Table 1:** EASE analysis.

Gene Category: Increased Expression	EASE score	Gene Category: Decreased Expression	EASE score
intracellular transport	0.002	chromatin remodeling	0.006
neuronal cell recognition	0.005	non-covalent chromatin modification	0.006
vesicle-mediated transport	0.007	response to chemical substance	0.012
transport	0.010	regulation of cell cycle	0.015
synaptic transmission	0.013	chromatin modification	0.040
transmission of nerve impulse	0.017	alcohol metabolism	0.051
glucose catabolism	0.029	carboxylic acid metabolism	0.053
cell recognition	0.037	cell cycle	0.063
intracellular protein transport	0.037		
Golgi vesicle transport	0.040		
hexose catabolism	0.042		

Using EASE (http://david.abcc.ncifcrf.gov/), genes with increased expression in IAD are significantly overrepresented in gene ontology biological process categories that are important for synaptic function such as synaptic transmission, neuronal cell recognition and vesicle-mediated transmission. Genes with decreased expression in IAD are significantly overrepresented in categories relevant to chromatin modification, cell-cycle and carbohydrate metabolism. EASE score equals the upper bound of the distribution of Jacknife Fisher exact probabilities given the List Hits, List Total, Population Hits and Population Total. Upregulated genes (303) and downregulated genes (157) were separately analyzed with EASE.

### Neuroplasticity and synaptic terminal function

To augment the results of EASE analysis we analyzed the genes in Table S2 (p<0.01) with the web-based Ingenuity® Pathway Analysis software (Ingenuity® Systems, http://www.ingenuity.com). The functional analysis identifies the biological functions and/or diseases that were most significant to the data set (Figure S3). As the two most significant categories contain many of the same genes, we chose to focus on genes with expression changes of 1.4 or greater within the larger category “Nervous system function and development” (−log4 significance) (Table 2, and for the complete list Table S3), which includes *GluR2* (designated *GRIA2*) and the glutamate transporter 2 (*SCL1A2*), both relevant to neuroplasticity. The muscarinic acetylcholine and serotonergic neurotransmitter receptors are also of interest. Increased expression of *GABRA1*, *GABRA2 and GABBR2* suggests activation of GABAergic inhibitory pathways. We also generated networks of the focus genes (p<0.01 group) which were algorithmically generated from information contained in the Ingenuity Pathways Knowledge Base based on their connectivity (Table S4). Network #3 (Figure S4A) contains 37 genes involved in neurological disease, 22 of which are overexpressed in IAD and Network #4 (Figure S4B) contains 26 genes contributing to nervous system function and development, 17 of which are overexpressed in IAD (Figure S4). Including genes which interact with GluR2 in Network 4 demonstrates the potential repercussions of changes in expression levels of one gene (Figure S4C).

**Table 2:** Nervous system function and development genes upregulated with ≥1.4 fold change in IAD.

GENE SYMBOL	GENE NAME	Fold change
CHRM3	cholinergic receptor, muscarinic 3	2.3
GRIA2	glutamate receptor, ionotropic, AMPA 2 (GluR2)	2.3
NRGN	neurogranin (protein kinase C substrate, RC3)	1.9
SLC1A2	solute carrier family 1 , member2 (GLT2)	1.9
HOMER1	Homer homolog 1 (Drosophila)	1.7
EPHA4	EPH receptor A4	1.8
HTR2A	5-hydroxytryptamine (serotonin) receptor 2A	1.8
THY1	Thy-1 cell surface antigen	1.8
NRXN1	neurexin 1	1.7
GPR51	G protein-coupled receptor 51	1.7
CAMK2B	Calcium/calmodulin-dependant protein kinase II beta	1.7
RAB14	RAB14 member RAS oncogene family	1.6
APOE	apolipoprotein E	1.5
SV2A	synaptic vesicle glycoprotein 2A	1.5
GAP43	growth associated protein 43	1.5
SCN1B	sodium channel, voltage-gated, type I, beta	1.5
ARNT2	aryl-hydrocarbon receptor nuclear translocator 2	1.5
PRKCZ	protein kinase C, zeta	1.5
LPPR4	Plasticity related gene	1.5
PUM1	Pumilio homolog 1 (Drosophila)	1.4

The Ingenuity® category ‘Nervous System Development and Function’ contains many genes involved in synaptic function, including those that are identified by EASE analysis. Many of these genes encode putative mRNA binding sites for proteins which regulate translation (see Table S5 for information on 3′UTR regulatory sequences).

### Local translation at the synapse

Increased expression of mRNA species translated at the synapse may impact synaptic function in IAD. The 3′ UTR of each gene with changed expression in IAD was analyzed for four potential consensus sequence sites regulating translation; the cytoplasmic polyadenylation element (CPE) [44]–[46], the fragile X mental retardation 1 (FMRP) binding G-quartet [47], the AU-rich element which binds to the mRNA binding protein, HuD [48], and a putative pumilio binding site [49]. As seen in Table S5, 24 of 49 encode at least one of these putative regulatory sequence(s) in the 3′UTR, and 10 of these contain more than one regulatory sequence. Twenty-five mRNAs do not contain any of the consensus sequences although two of these genes, *GAP-43* and neurogranin *(NRGN)*, important for neuroplasticity, are known to encode other regulatory elements.

### Expression of *GluR2* mRNA and corresponding protein

**Figure 4:**
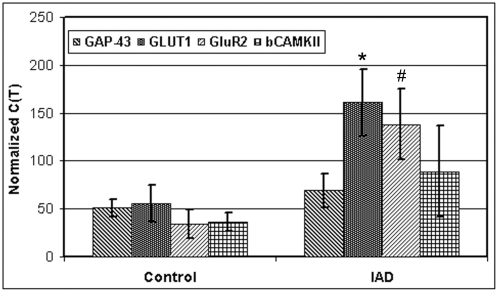
Quantitative RT-PCR confirms upregulation of GLUT1 and GluR2. Total RNA (2 µg) was used to generate cDNA by reverse transcription using oligo-dT primers. Note increased mRNA levels of glutamate transporter (GLUT1) and GluR2 in IAD patients compared to controls. Data are mean values ±SEM comparing 4 control and 4 IAD patients. βCAMKII and GAP43 do not show significant change in mRNA levels. GAP-3 (p<0.1), GLUT1 (* p<0.01), GluR2 (# p<0.03), βCAMKII (p<0.06).

To quantify the differential expression patterns determined by microarray, synaptoneurosome mRNA from 5 control and 5 IAD patients was tested by quantitative RT-PCR. Genes selected from the microarray data set represented a spectrum of function. Quantitative RT-PCR analyses confirmed the mRNA expression changes detected in the microarray between control and IAD patients (Figure 4) and indicate a higher fold change in IAD with, *GLUT1* (×3.0, p<0.01) and *GluR2* (×3.5, p<0.03).

**Figure 5:**
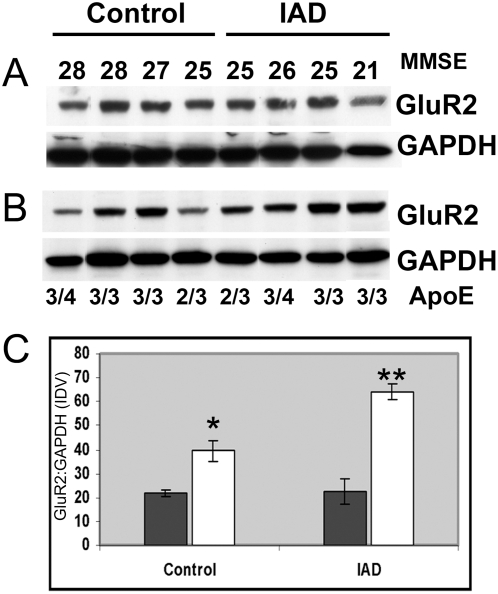
GluR2 protein expression. To determine if increased GluR2 mRNA generates elevated protein expression, we compared subjects with a range of MMSE values. Representative immunoblots of A) homogenates and B) synaptoneurosomes, (10 µg protein per lane) were detected with antibodies for GluR2 and GAPDH. C) In controls, the increased ratio (2-fold) of synaptoneurosome (white bar) GluR2 to total GluR2 in homogenates (* p<0.0002) is an indication of synaptic protein enrichment and this ratio increases by 50% in IAD patients (p<5.8E-08). In the IAD group there is a significant increase in synaptoneurosome GluR2 protein expression compared to control (** p<6.0E-05). Homogenate GluR2 (black bar) remains constant in controls and IAD. Data are mean values ±SEM for 4 controls and 4 IAD analyzed in 5 separate experiments. The ratio of GluR2 to GAPDH is given in arbitrary units based on the integrated density value which is the sum of all pixel values after background correction (IDV).

GluR2 was selected to determine if protein expression correlates with the increased mRNA seen in synaptoneurosomes from individual patients. Equal amounts of protein (25 µg) from homogenate and synaptoneurosome preparations were separated by polyacrylamide gel electrophoresis (PAGE). Immunoblots labeled with anti-GluR2 and GAPDH revealed that, in controls, there is a significant increase in the GluR2 subunit in synaptoneurosomes compared to whole homogenates (p<0.0002), confirming the data presented in Figure 1. In synaptoneurosomes from IAD patients, GluR2 increased as MMSE declined, whereas total GluR2 in homogenates remained relatively stable (Figure 5A, B, C). In Figure 5C, the ratio of synaptoneurosome-associated GluR2 to total GluR2 present in homogenates increases 2-fold in controls to more than 3-fold in IAD. The increase in synaptic GluR2 subunits is masked in homogenates. Thus, IAD patients have increased levels of GluR2 mRNA at the synapse which generate an increase in receptor subunits.

## Discussion

### Gene Expression in IAD Patients

Using mRNA isolated from synaptoneurosome-enriched preparations, we distinguished early molecular changes in the brains of IAD patients which, by current consensus, occur at the synapse [1], [50], [51]. We identified a panel of mRNAs with increased expression in IAD patients which are overrepresented in the gene ontology, biological process categories of synaptic transmission and transport. oAβ burden was concomitant with a decline in cognitive function even though these patients had minimal neuropathological changes in the prefrontal cortex. An increase in *GluR2* expression in IAD patients was accompanied by elevated expression in the corresponding protein.

Our results are in striking contrast to previous microarray studies, comparing either whole homogenates or single neurons from control and AD brains [26]–[29], which indicate increased expression in genes coding for inflammation, stress and DNA repair. Blalock et al [30] in an informative study focused on IAD compared hippocampal homogenates from 9 controls and 7 IAD subjects but found that expression values of genes overrepresented in the categories of synaptic transmission and cell-surface signal transduction were decreased whereas genes with increased expression were overrepresented in the category regulation of transcription. These discrepancies are not unexpected as our studies differ in several ways. For instance, we used prefrontal cortex which is affected pathologically after the hippocampus [52] and therefore might yield data on earlier molecular changes. Also, as the synapse is likely the site of initial Aβ toxicity, we chose to analyze genes directly impacted by synaptic events which are localized and translated at the synapse. It is possible that, in IAD, selective targeting and transport of some mRNAs to the synapse is modified but total mRNA is not appreciably altered. We found only 5 overlapping genes in the upregulated category of each study and no common downregulated genes. Nevertheless the results from these studies are complementary with our study focusing on molecular changes at the synapse and that of Blalock et al defining regional gene expression differences in IAD.

### Enrichment of synaptic mRNAs

Isolation of mRNAs from synaptoneurosomes provides a selective enrichment of a cohort of genes encoding synaptic terminal proteins locally translated in the synapse. Expression profiles of genes from whole homogenates of human CNS tissue reflect a bias in the ratio of whole cell to synaptic mRNA. Thus, even though our preparations may contain some nonsynaptic, cellular components [53], [54], the enrichment of synaptic mRNAs enables detection of expression changes in genes, such as GluR2, at their sites of translation in the synapse, that are masked by more abundant mRNAs in whole brain homogenates. Our results also substantiate the prospect of using postmortem human CNS tissues for functional analysis as described for tissue slices by Hahn [21]. The *in vitro* protein synthesis assay demonstrates the functional stability of synaptoneurosomes obtained postmortem providing an assessment of basal levels of *in vitro* protein synthesis and the potential to investigate receptor-based regulation of mRNA translation at the synapse in neurodegenerative diseases such as AD.

### Dementia progression in IAD and compensatory neuroplasticity in the prefrontal cortex

Our microarray profiles positively correlate with global indicators of dementia progression and display a high specificity and significance for patients with MMSE scores consistent with IAD. Measures of frontal lobe executive function show no statistically significant differences in the composite measure of Digit Span Backwards and neuropathological changes are minimal to absent. Thus, microarray and protein changes are detectable in the prefrontal cortex prior to overt behavioral and neuropsychological deterioration. Executive functioning is not a unitary construct however, but can be fractionated into multiple component parts and includes the ability to focus and shift attention, to inhibit responses, to think abstractly, and to solve problems [55]. There was a trend for impaired recall in IAD patients which may correlate with hippocampal pathology, as indicated by Braak scores up to III. Thus, in contrast to molecular profiles, use of tests that measure executive functions other than working memory cannot definitively differentiate the two patient groups. Alternatively, our small sample size likely lacked sufficient power to detect group differences in executive functioning.

In contrast to the decline in cognitive function, our microarray expression profiles of IAD patients reveal a seemingly paradoxical increase in mRNAs encoding proteins which regulate synaptic transmission, plasticity and signal transduction. These results are not without precedent. Firstly, a morphometric analysis of antibody staining in brain sections of AD patients up to Braak stage III reveals an increase in the presynaptic proteins SNAP-25, synaptophysin, syntaxin and α-synuclein [56]. With further disease progression, when neocortical neuropathologic changes of AD appear, the increase is reversed (data not shown) in accord with other reports comparing control and AD brains which show a general decrease in synaptic proteins in moderate to severe AD [57]. Two other synaptic proteins, drebrin, which mediates synaptic plasticity by regulating spine morphogenesis and receptor density on spines [22], and the vesicular glutamate transporter 1 (VGluT1), a presynaptic transporter protein are also elevated in IAD patients [58], [59].

These findings dovetail with reports of compensatory plasticity which is mediated, in part, by ApoE [60]. Increased expression of *ApoE* mRNA in IAD patients, along with other plasticity-related genes, such as growth-associated protein 43 (*GAP-43*), plasticity related gene (*LPPR4*), *NRGN*, synaptogyrin (*SYNGR1*) and beta-synuclein (*SNCB*) suggest a concerted response. Compensatory plasticity is further supported by brain functional imaging which reveal distinct cortical activation patterns suggesting the brain might substitute alternative pathways to maintain successful task performance in IAD when confronted with impaired access to specific task-subserving areas, [61], [62]. Functional activation studies comparing AD and healthy, aging brains [63], [64] also reveal very specific cortical activation patterns, which correlate with performance benefits. Increased expression of two mRNAs encoding gamma-aminobutyric acid (GABA-A) receptors *(GABRA1 and GABRA2)* and one GABA-B receptor (*GABBR2*) in IAD patients, may indicate GABAergic terminal sprouting and suggests that inhibitory compensatory mechanisms may also be activated, perhaps due to aberrant increases in network excitability as in the hAPP transgenic mouse [65].

### dAβ accumulate as MMSE declines

The dimeric form of Aβ1-42 becomes detectable on Western blots of the prefrontal cortices of our IAD patients, and further increases with advancing dementia. Our data (Figure S3) suggests that oligomers, other than the dimer, do not show accumulation concurrent with dementia. In experimental mouse models, Aβ dimers or trimers are implicated and produce transient brain dysfunction in the absence of plaques detected neuropathologically [14]. Accumulation of dAβ in Tg mice also occurs concurrently with memory impairment [12]. In experiments using extracts from AD brains, dAβ is shown to have a primary role in impairment of plasticity in rodent slice cultures and to disrupt memory function in rats trained on a step-through passive avoidance task [66].

We found that dAβ is more abundant in whole homogenates than in synaptoneurosome fractions suggesting only a small portion of total dAβ is associated with synaptic membrane or present in the postsynaptic compartment [3], [12]. The prefrontal cortices of two control and three IAD patients have some amyloid plaque accumulation (Table S1), but Aβ1-42 is reported in susceptible neurons of IAD patients even before plaque pathology is apparent. dAβ is not detected in control patients except for one individual (Figure 2) with an *ApoE3/4* genotype but normal MMSE and CDR scores. Increased *ApoE* mRNA expression in IAD patients (Table 2), may augment the effects of the *ApoE4* allele, a major genetic risk factor for AD that leads to excess amyloid buildup in the brain before AD symptoms arise.

### Synaptic mRNA and local translation

The *de novo* synthesis of proteins in response to receptor activation is an essential component of plasticity effecting immediate changes in the balance of local constituent proteins at the synapse [33], [67]–[69]. Many of the mRNAs with the greatest fold change in our study encode putative mRNA binding sites in the 3′UTR which regulate local protein synthesis through interacting binding proteins (Table S5). In fact, our results may underestimate the percentage of mRNAs encoding regulatory site as not all potential regulatory sites were examined. For instance, mRNA for the atypical brain specific isoform of protein kinase C zeta (*PKMζ*) is transported to dendrites and co-localizes with *BC1* mRNA, a translational repressor [70]. Nevertheless, these results support the strategy of isolating synaptoneurosomes for boosting synaptic terminal mRNAs revealing expression changes previously undetectable in whole homogenates.

*GluR2* mRNA is present in a high proportion of dendrites in the frontal cortex, as it is in hippocampal neurons, [71] and is targeted for activity-dependant protein synthesis, [69], [72] Three putative consensus sequences encoded in *GluR2* mRNA, specifically, a CPE-like binding sequence [73], a G-quartet and a putative pumilio binding domain (Table S3) provide potential diverse and versatile mechanisms for regulation of *GluR2* translation. CPE-like consensus sequence sites are crucial for dendritic plasticity and thus for learning and memory [33]. As yet, only one mechanism has been determined for regulation of *GluR2* translation, mediated via Type 1 metabotropic glutamate receptors (mGluRs) [72]. In accord with this, we found that *GluR2* encodes a G-quartet sequences in the 3′UTR (Table S5) and a preceding G-rich sequence consistent with this mechanism [47], [74], suggesting that *GluR2* is a potential target of FMRP. Putative G-quartet structures were encoded by several other mRNAs, although regulation by FMRP has not been demonstrated for these genes.

Two mRNAs encoding RNA-binding proteins, HuD and the pumilio homolog 1, showed increased expression in IAD. Our study did not reveal a change in expression of *CPEB*1 and *FMR1*, the most studied regulators of protein synthesis at the synapse.

### Aβ and *GluR2* mRNA regulation in IAD

Our results indicate increased mRNA expression of several neurotransmitter receptors in IAD with the *GluR2* subunit of the AMPA receptor and the muscarinic cholinergic receptor 3 *(CHRM3)* showing the greatest change. Feedback on transcription may contribute to an increase in postsynaptic receptor mRNA. In primary rat hippocampal cultures, NMDAR signaling inhibits transcription and decreases *GluR1* and *GluR2* mRNA in dendrites [71]. Speculatively, as Aβ binding to the α-7-nicotinic acetylcholine (ACH) receptor induces NMDAR endocytosis [9] thereby reducing NMDA receptor activation and signaling, disinhibition of *GluR2* gene transcription may follow. Consequently, a larger pool of somal *GluR2* mRNA may be available for subsequent targeting and transport to dendrites in response to signaling via Group 1 mGluRs, which are known to promote a transcription-independent increase in dendritic *GluR* s through transport from the soma [71]. Although the mechanism is currently uncertain our data suggests that in IAD there is an increase in transport of some mRNAs to the synapse.

### Muscarinic acetylcholine receptor M3

Several studies have suggested that targeting muscarinic receptors in AD patients may have therapeutic benefit. For instance, agonist stimulation of the M1 and M3 receptors in human cell cultures increases APP release but downregulates the amyloidogenic pathway leading to Aβ production [75], [76]. In accord, rats treated with a muscarinic agonist show decreased levels of APP in neocortex, hippocampus and striatum along with a decrease in Aβ suggesting an enhancement of the non-amyloidogenic pathway [77]. The neuroprotective effect of muscarinic transmission is also seen in studies with the 3×Tg-AD mouse model of AD in which, after treatment with an M1 muscarinic agonist, mice showed a decrease in cognitive decline and an accompanying reduction in Aβ and tau pathology [78]. Since activation of the M3 receptor has similar effects on APP processing, increase in M3 mRNA may be a compensatory mechanism in IAD patients responding to Aβ overproduction.

### GluR2-interacting molecules: inhibitory and compensatory mechanisms?

Previous studies on GluR2-interacting proteins which, in our study, are overexpresssed in IAD, lend credence to the notion that both inhibitory and compensatory mechanism are driven by these genes. For instance, adenatomous polyposis coli (APC) which is highly expressed in developing brain is required for AMPAR activity, facilitating clustering of both AMPARs and PSD-95 at the synapse and inducing AMPAR activity [79], [80] . Also PKMζ, an atypical protein kinase C isoform, regulates movement of AMPARs to the synapse and maintains them in late LTP [81], [82]. Notably though, PKMζ does not play a role in the establishment of short-term memory [82] which is most at risk in incipient and early AD.

Overexpression of other genes, may have a negative effect on AMPAR mediated excitatory currents. The short splice variant of Homer, 1a, which is expressed in response to synaptic activity, reduces surface AMPARs and depresses AMPAR postsynaptic currents [83]. Thus an increase in *Homer* mRNA, might result in higher levels of Homer 1a, perhaps in response to the excitotoxic effects of oAβ in IAD patients, intensifying the negative feedback, activity dependant loop regulating the structure and function of synapses. However, to our knowledge, the effect of oAβ on Homer expression has not been investigated. Dynamin also regulates removal of AMPARs from the membrane and GluR2 undergoes constitutive and ligand-induced internalization that requires the clathrin adaptor complex AP-2 in addition to dynamin [84], [85].

### GluR2 subunit regulation, oAβ and neuroplasticity

The accumulation of toxic Aβ oligomers in IAD is particularly relevant to GluR- mediated plasticity as Aβ perturbs basal glutamatergic synaptic transmission in rat cortical slices by a selective reduction in postsynaptic, AMPAR-mediated currents [86]. Our data suggests that the increase in synaptic *GluR2* results in a corresponding increase in protein expression at the synapse in IAD patients. The GluR2 subunit defines the properties of AMPARs which are heteromers, consisting mainly of GluR1 and GluR2 subunits in the mature brain [87]. GluR2 insertion maintains the receptors in a low conductance state and NMDAR activation is required to induce a rapid incorporation of GluR1, forming Ca^++^ permeable AMPAR homomers which are required for LTP [88]. Although we show an increase in relative amounts of synaptoneurosome-bound GluR2 subunits in IAD compared to control, we do not yet know if they are synaptic, extrasynaptic or internal. However it is reported that, in primary cultures of rat hippocampal neurons, chronic treatment with Aβ diffusible ligands induces an aggregation of GluR2 on the surface of dendrites [89] as well as driving the loss of surface GluR1 subunits by endocytosis leading to LTD [90], and ultimately, loss of dendritic spines and synapses. Impeding the influx of Ca^++^ into neurons through insertion of GluR2 into AMPARs may be a response to dAβ that is initially neuroprotective but which eventually leads to synaptic depression, and in AD patients, cognitive impairment.

These findings may provide targets for pharmaceutical intervention, either to moderate negative impact of Aβ on cognition via GluR2 or to stimulate compensatory activity. Also our approach using synaptoneurosomes from human brain may be useful for elucidating molecular abnormalities at the synapse in other diseases of the brain such as Parkinson's disease and schizophrenia.

## Materials and Methods

### Disclosure statement

Approval for patient neuropsychological testing (IRB approval # 002003) and for harvesting of the brain at autopsy (IRB approval # 042071) was obtained from the USC Health Sciences Institutional Review Board. Written informed consent was obtained from all participants during an interview conducted by the Clinical Core of the ADRC in which patients were invited to enroll in the study.

### Neuropsychological and Neuropathological Assessment

Neuropsychological and Neuropathological Assessment of the control and IAD patients are explained in Supplemental Methods S1.

### Preparation of Synaptoneurosomes

Blocks (1 cm^3^) were obtained from prefrontal cortices (Brodmann's areas 9, 10) at autopsy from a total of 14 patients; 8 controls, and 6 IAD, immediately snap-frozen and stored at −90°C. Four samples, 2 controls and 2 IADs, with additional available tissues were re-analyzed as duplicates for microarrays. Synaptoneurosomes were prepared by a standard method with slight modifications [35], [37], [74], [91]. Frontal cortex (0.5 gm–2.5 gm) was thawed and homogenized with a Teflon-homogenizer (4 strokes at 1000 rpm) in buffer (1/10wt/vol), containing 0.35 M sucrose pH 7.4, 10 mM 4-(2-hydroxyethyl)-1-piperazineethanesulfonic acid (HEPES), 1 mM ethylenediaminetetraacetic acid (EDTA), 0.25 mM dithiothreitol, 30 U/ml RNAse inhibitor and a protease inhibitor cocktail (Pierce, Rockford, Il). Cell debris and nuclei were removed by centrifugation at 1000 g for 10 min at 4°C yielding pellet P1 and supernatant S1. The S1 fraction was passed sequentially through a series of screens with decreasing pore sizes of 100, 80, 30 and 10 µm. The final filtrate was resuspended in 3 volumes of buffer without sucrose and centrifuged at 2000×g, for 15 minutes at 4°C to yield a pellet containing synaptoneurosomes. Total preparation time did not exceed 1 hour. For detection of nuclear contamination, 5 µl of homogenate (2 ml total volume), P1 (0.5 ml) or synaptoneurosome pellets (0.1 ml total volume) were smeared and air-dried onto microscope slides then fixed in ice-cold acetone for 5 minutes. Nuclei were labeled by addition of mounting media containing DAPI. Representative fields at 20× magnification from homogenate, P1 and synaptoneurosome fractions were counted to assess nuclear content. Synaptoneurosome pellets were snap-frozen for mRNA preparation or suspended in incubation buffer. Some samples were prepared for electron microscopy by fixation in 1% paraformaldehyde/0.1% glutaraldehyde, postfixed in 1% osmium tetroxide, washed in phosphate buffer then further processed as detailed by Johnson et al (1997).

### Total RNA isolation and microarray preparation

Total RNA from synaptoneurosome preparations was extracted with Trizol LS (Invitrogen Carlsbad, California 92008) and purified with RNeasy columns (Qiagen, Valencia CA 91355). The RNA was quantified and checked for purity by comparison of absorbance at 260 and 280 nm in the Nanodrop Spectrophotometer (Nanodrop, Wilmington, DE 19810). Total RNA and mRNA were analyzed for integrity and concentration by microanalysis in an Agilent bioanalyzer (Agilent Santa Clara, CA 95051). Probes for array analysis were prepared according to the Affymetrix protocol using 10 µg total RNA as a template to generate cDNA probes for hybridization. Biotinylation and amplification of cDNA was accomplished by the use of an Enzo BioArray High Yield RNA Transcript Labeling Kit (Enzo Life Sciences, New York,NY) followed by purification by absorption over an RNeasy column (Qiagen). Hybridization to the HG-U133A (Affymetrix) was performed according to standard Affymetrix protocols. The hybridized array was washed, labeled with phycoerythrin-conjugated streptavidin (Molecular Probes, Eugene, OR) and scanned in an Affymetrix scanner.

### Data analysis

Microarray Analysis Suite 5.0 (MAS 5.0) was used for the initial signal analysis which generates a signal detection value based on the difference between the perfect match (PM) and mismatch (MM) probes in a probe set. A ‘present’ or ‘absent’ call was assigned to each gene by MAS 5.0 according to certain threshold and the detection p-values. The microarray data was checked for RNA degradation, visible defects in images, and Affymetrix hybridization control by Bioconductor Affymetrix package (http://www.bioconductor.org/) [92], [93]. Model-Based Expression Index (dChip) [94] implemented in Bioconductor, was used for signal normalization within an individual chip and across all samples. For high-level analysis, statistics tests were conducted to find significantly expressed genes, and this small set of genes was applied for clustering and pathway analysis. Probe sets that were differentially expressed among control and IAD were identified using ANOVA. Benjamini and Hochberg's False Discovery Rate (FDR) was used for adjusting for multiple testing. The genes with significant change in expression were subject to hierarchical clustering using dChip. Gene annotation and gene ontology grouping was identified with the Expression Analysis Systematic Explorer or EASE (http://david.abcc.ncifcrf.gov/ ) for assignment of significantly expressed genes to Gene Ontology Consortium categories (http://www.geneontology.org). Functional grouping of genes and pathways were further explored with Ingenuity Pathway Analysis, (Ingenuity® Systems http://www.ingenuity.com). The p<0.01 dataset containing gene identifiers and corresponding expression values was uploaded into the application. Each identifier was mapped to its corresponding gene object in the Ingenuity knowledge base. For this analysis a fold change of ≥1.2 was set to identify genes whose expression was significantly differentially regulated. These genes, called focus genes, were overlaid onto a global molecular network developed from information contained in the Ingenuity knowledge base. Networks of these focus genes were then algorithmically generated based on their connectivity.

The 3′UTR CPE-element important in mRNA processing at the synapse was identified using UTRScan at http://bighost.ba.itb.cnr.it/BIG/UTRScan/ [46] and AU-rich sequences were identified using the search tool at http://brp.kfshrc.edu.sa/ARED/ A further analysis, using ESPSearch [95] was carried out for mRNAs bearing CPE-like sequences using broadened criteria. [45]. A detailed explanation of 3′UTR analysis is explained in Supplemental Methods S1.

### Independent validation of the microarray data by real time quantitative PCR

Genes of interest and showing significant alterations in expression were used for RT-PCR analysis with gene-specific amplification primers to confirm the array results. β-2-microglobulin (β-2M) probe was used as a normalization control. Briefly, 2 µg total RNA was used to generate cDNA by reverse transcription using oligo dT primers. Subsequently, the cDNA product was used for the quantitative PCR reaction in the DNA Engine Opticon 2 Continuous Fluorescence Detection System (BioRad) using SYBR Green chemistry with the DyNamo HS kit (New England Biolabs, Ipswitch, MA). Evaluation samples were run in triplicate with 10-fold serial dilution. Signal intensity to cycle number was monitored during the run. The differential mRNA expression of each studied gene was calculated with the comparative Ct method using the formula 2^ΔΔCt^ where ΔCt stands for the difference between the target gene and the endogenous control β-2M, adjusted by the Ct difference between these 2 genes in negative controls, and ΔΔCt equals to the difference between the ΔCt value of the target gene in samples.

### *In vitro* translation

Two reactions were set up using mRNAs isolated from synaptoneurosomal preparations of pooled human normal controls or pooled AD-affected frontal cortex, and containing exogenous cellular components, the rabbit reticulocyte lysate (RRL) necessary for protein synthesis. Each reaction contained RRL (37 µl); (Promega; Madison,WI), nuclease free water (10 µl), 1 mM amino acid mix (1 µl), RNAsin (1 µl), RNA template (2 µl); The components were thoroughly mixed before addition of Transcend tRNA (2 µl); (Promega; Madison,WI). A no-template control was also included to distinguish endogenous biotinylated proteins from newly synthesized translation product. To establish that preparations retained independent basal translation capability, reactions containing synaptoneurosomes but not RRLs were tested. Each reaction contained synaptoneurosomes (10 µg protein in 37 µl translation buffer) from a control patients brain, nuclease free water (10 µl),1 mM amino acid mix (1 µl), RNASin (1 µl) and Transcend t-RNA(2 µl). Control reactions contained either the translation inhibitor anisomycin (40 µM) or the transcription inhibitor actinomycin D (5 µg in 50 µl). Five further reactions were set up as described with synaptoneurosomes from 3 controls and 2 MCI patients and incubated for 40 minutes at 30°C to determine the repeatability of the translation capability in synaptoneurosomes from human brain autopsy tissue. The reactions were incubated for 40 minutes at 30°C and 10 µl applied to gels for detection of biotinylated proteins on Western blots with streptavidin-horseradish-peroxidase conjugate (GE Healthcare, Piscataway, NJ) followed by Supersignal West FEMTO chemiluminescent substrate.

### Immunoblots

Enrichment and stability of proteins in homogenates and synaptoneurosomes were analyzed on Western blots with the following antibodies: anti PSD95 (MAB1596), NMDAR1 (MAB363)., and GluR2 (AB1768-25UG ), SNAP25, and GAPDH (MAB374) all from Chemicon; Temecula, CA. βIII Tubulin (Abcam; Cambridge MA) and PKCα,and GFAP (Santa Cruz Biotechnology; Santa Cruz, CA) were used to detect cytoplasmic and glial proteins. MAb 4G8 (Abcam, Cambridge,MA) was used for Aβ peptide detection. Samples were separated on 5–15 or 10–20% gradient tris-glycine polyacrylamide gels and electrotransferred to nitrocellulose at 100 V for 2 hours. Membranes were incubated overnight at 4°C with primary antibodies and then with horseradish-peroxidase-linked (HRP) secondary antibodies (Pierce; Rockford, IL) for 1 hour. The signal was detected with ECL reagent (GE Healthcare; Piscataway, NJ).

### Densitometry and Statistical Analysis

Immunoblots were scanned and assessed by densitometry with AlphaEase™ v5.5 software. The ratio of GluR2 or dAβ to GAPDH is given in arbitrary units based on the integrated density value which is the sum of all pixel values after background correction (IDV). Values are presented as mean±SEM.. Comparisons were made between control and IAD using ANOVA. A p-value of less than 0.05 was considered statistically significant.

## Supporting Information

Supplemental Methods S1 (0.09 MB PDF)

Table S1 Neuropsychological and Neuropathological Assessment of Patients. The IAD group and the normal control group were not statistically different with regard to age, years of education, gender distribution, or interval between testing and death. As expected, the IAD group obtained lower scores on the MMSE than normal controls (p<0.01). MMSE scores ranged from 25 to 29 for normal controls and from 21 to 27 for IAD. No statistically significant difference was detected between NC and IAD for performance on either the composite measure or Digit Span backward. Statistical comparisons on each measure were made by Mann-Whitney U tests.
(0.02 MB PDF)

Table S2 Differentially Expressed Genes in IAD (p<0.01) with fold change, p-values and RefSeq Summary.
(0.21 MB XLS)

Table S3 Ingenuity Functional Analysis. Genes comprising the two most significant categories, Cell-to-Cell Signaling and Interactions and Nervous System Function and Development.
(0.04 MB XLS)

Table S4 Network representation of the biological processes generated by genes upregulated in IAD. The data set containing gene identifiers and corresponding expression values for the overexpressed C v IAD genes in the p<0.01 group were uploaded and overlaid onto a global molecular network developed from information contained in the Ingenuity Pathways Knowledge Base. Each gene identifier was mapped to its corresponding gene object in the Ingenuity Pathways Knowledge Base. Networks of these focus genes were then algorithmically generated based on their connectivity. Our genes fell into 24 Ingenuity defined networks.
(0.02 MB XLS)

Table S5 Regulatory elements present in the 3′UTR of genes important for nervous system function and development. The 3′UTR of each gene with changed expression in IAD was analyzed for four potential consensus sequence sites regulating translation; the cytoplasmic polyadenylation element (CPE), the FMRP binding G-quartet, the AU-rich element which binds to the mRNA binding protein HuD, and a putative pumilio binding site. At least one of these putative regulatory sequence(s) is encoded in the 3′UTR of 24 out of 49 total genes and 3 and 10 of these contain more than one regulatory sequence. Twenty-five mRNAs do not contain any of the consensus sequences although two of these genes, GAP-43 and neurogranin, important for neuroplasticity, are known to encode other regulatory elements. Reported genome content of the regulatory sequences is included at the bottom of the relevant column.
(0.31 MB XLS)

Figure S1 Functional Stability of Synaptoneurosome mRNA. A)To test stability of mRNA from synaptoneurosomes, pooled mRNAs from control tissues were combined with rabbit reticulocyte lysates (RRL) and Transcend™ tRNA, which is an ε-labeled, biotinylated lysine-tRNA complex with a detection sensitivity of 0.5–5 ng of protein, and the resulting biotinylated proteins detected by Western immunoblot. In combination with rabbit reticulocyte lysate (RRL) and Transcend™ biotinylated t-RNA the isolated mRNAs from control (lane 2) and AD tissues (lane 3) yielded several biotinylated species indicative of newly synthesized proteins seen between 70–90 kD (two bands), ∼60 kD and ∼50 kD, and two bands >30 kD. The identity of these proteins is undetermined. A no-template control, with RRLs only, shows that bands at ∼70D (also in Control and AD lanes) and 30 kD are endogenously biotinylated proteins (asterisks). B) In vitro translation function of synaptoneurosomes is maintained in the 5 patients tested, regardless of clinical stage of disease. Asterisks denote endogenously biotinylated species.
(0.20 MB PDF)

Figure S2 Immunoblots of Homogenates and Synaptoneurosomes Probed with MAbs 4G8 and 6E10. A) Only the band coinciding with dimeric Aβ (∼9 KD, arrowhead)is correlated with declining MMSEs or ApoE genotype in homogenates or synaptoneurosomes. B) Densitometry reveals that, when normalized to GAPDH, dimeric Aβ but not tetrameric Aβ levels are inversely related to MMSE. Because of small n and variability within groups, differences between groups is not significant . dAβ C v IAD p = 0.35, C v AD p = 0.12. tAβ C v IAD p = 0.99, C v AD p = 0.44 C) Immunoblots of control (lane1), IAD (lane2) and AD patients (lane 3,4) probed with antibodies 6E10 and 4G8 revealed labeling of dAβ only in patients with cognitive decline.
(0.10 MB PDF)

Figure S3 Ingenuity Functional Analysis. The Functional Analysis identifies the biological functions and/or diseases that were most significant to the data set. Genes from the p<0.01 dataset with fold change ≥1.2 in IAD patients were used for the analysis. Fischer's exact test was used to calculate a p-value determining the probability that each biological function assigned to that data set is due to chance alone. Threshold is at 1.3 = −log (p<0.05).
(0.03 MB PDF)

Figure S4 Network representation of the biological processes generated by genes upregulated in IAD. The Neurological Disease Network contains 37genes, 22 of which are focus genes upregulated in IAD (Figure S4A). Increased expression of two mRNAs encoding gamma-aminobutyric acid (GABA-A) receptors (GABRA1 and GABRA2) and one GABA-B receptor (GABBR2) in IAD patients, may indicate GABAergic terminal sprouting and suggests that inhibitory compensatory mechanisms may also be activated The Nervous System Development and Function Network (Figure S4B) contains 26 genes 17 of which are upregulated in IAD. In Figure S4C, Network 4 in subcellular layout, the functional significance of GluR2 (GRIA2) is seen by the addition of up- and downstream interacting molecules to the network. Notably, GluR2 is a potential target of FMRP. Other significant GluR2-interacting molecules, dynamin, AP-2 and Homer, are not represented in this chart. Genes overexpressed in IAD are seen in pink with fold change. All networks are listed in Table S4.
(0.71 MB PDF)
