# Supplementary material for: Transcriptome Analysis of Synaptoneurosomes Identifies Neuroplasticity Genes Overexpressed in Incipient Alzheimer's Disease
Source: PLoS One. 2009 Mar 19;4(3):e4936. doi: 10.1371/journal.pone.0004936 (PMC2654156; doi:10.1371/journal.pone.0004936)
Supplement: Supplemental Methods S1 — (0.09 MB PDF) [file pone.0004936.s001.pdf]

Transcriptome Analysis of Synaptoneurosomes Identifies Neuroplasticity Genes Overexpressed in Incipient Alzheimer's Disease

Celia Williams, Ruty Mehrian Shai, Yongchun Wu, Ya-Hsuan Hsu, Traci Sitzer, Bryan Spann, Carol McCleary, Yi Mo and Carol A. Miller

Supplemental Methods and References

Patient information:

CNS tissues were obtained from the USC Alzheimer's Disease Research Center (ADRC) Neuropathology Core (NIA AG05142)(IRB approval # 042071). Clinical information, including neurological examination, neuropsychological testing such as cognitive assessment, family history, and medications were provided by the USC ADRC Clinical Core (IRB approval # 002003). The groups did not differ significantly in age or years of education. Medication histories indicated one IAD patient had used a selective serotonin uptake inhibitor. Postmortem intervals (PMIs) ranged from 2 to 9 hours (mean 5.1 hrs). Written informed consent was obtained from all participants for neuropsychological testing and for harvesting of the brain at autopsy.

Neuropsychological Assessment:

Each participant was tested individually by a trained psychometrist under the supervision of a licensed clinical neuropsychologist. Test scores from the evaluation closest to death were used in these analyses. Sample size varied slightly for some outcome measures due to missing data. Most cognitive evaluations were performed annually and, in most cases, less than one year prior to death. Two global indicators of cognitive status were used for clinical assessment of patients, the Clinical Dementia Rating (CDR) and the Mini Mental Status Examination (MMSE). For one patient, who was not assessed within two years of death, the MMSE was reduced from 27 to 25. Frontal lobe function was evaluated using measures of executive functioning, which included Digit Span backwards from the Wechsler Adult Intelligence Scale – Revised [1] and a composite measure comprised of items from the Mini Mental Status Exam (spell WORLD backwards) and the Short Blessed Test (say the months of the year in reverse order). Raw scores ranged from 0 to 7 on both measures and were assessed with the short or long version of the California Verbal Learning Test – second edition. These measures were administered as part of a larger neuropsychological battery designed to assess multiple cognitive domains.

Neuropathological Assessments

For neuropathologic diagnoses, modified CERAD [2] were used as well as the NIA/Reagan protocols. This includes Aβ burden and neurofibrillary pathology (NFT) assessments in multiple brain areas. For experimental studies, samples were obtained from the prefrontal cortex (Brodman's areas 9 and 10). Amyloid plaques and tangles were evaluated using the thioflavin-S fluoroscein and Gallyas silver stains in formalin-fixed, paraffin-embedded tissues taken from sites adjacent to that used for synaptoneurosome preparations. Scoring was performed by independent observations of three neuropathologists and an arbitrary score, reflecting amyloid or NFT burden as an average of all three readings (0=none, 1=sparse, 3=moderate, 5=frequent), was assigned to each individual. Braak scores assessing disease progression [3] and last date of neuropsychological testing were taken into account for borderline cases. Patients with Parkinson's disease related pathology were excluded.

3'UTR analysis (Table S5)

The 3'UTR CPE-element important in mRNA processing at the synapse was identified using UTRScan at http://www.ba.itb.cnr.it/UTR/ [4] and AU-rich sequences were identified using the search tool at http://rc.kfshrc.edu.sa/ARED/New_ARED.htm [5].

A further analysis, using ESPSearch was carried out for mRNAs bearing CPE-like sequences using a broadened criteria since, although the consensus CPE sequence is UUUUUAU, it has been reported that the sequence UUUUAU is also functional [6]. To generate our list of putative CPE bearing mRNAs we used targets with CPE upstream from the polyA signal defined by a study which identified CPEs in mouse cerebellar synaptoneurosomal fractions [7] but we allowed a distance of 200 base pairs between them as follows: TTTTTAT[5:201]AATAAA, TTTTTAT[5:201]ATTAAA, TTTTTAAT[5:201]AATAAA, TTTTTAAT[5:201]ATTAAA). Targets were compared to source cDNA sequences from genes of interest obtained at Entrez Nucleotide at http://www.ncbi.nlm.nih.gov/sites/entrez?db=nuccore&itool=toolbar . Putative G-quartet motifs and Pumilio binding sequences were identified in ESPSearch [8] using the respective consensus sequence DDGG[0:2]DDGG[0:2]DDGG[0:2]DDGG [9,10] and the ten nucleotide core motif [UGUA(A_C_U)AUA] [11]

References

1. Bak JS GR (1981) A review of the performance of aged adults on various Wechsler Memory Scale subtests. J Clin Psychol 37: 186-188.
2. Mirra SS, Heyman A, McKeel D, Sumi SM, Crain BJ, et al. (1991) The Consortium to Establish a Registry for Alzheimer's Disease (CERAD): Part II. Standardization of the neuropathologic assessment of Alzheimer's disease. 41: 479-.
3. Braak H, Braak E (1991) Neuropathological stageing of Alzheimer-related changes. Acta Neuropathologica 82: 239-259.
4. Mignone F, Grillo G, Licciulli F, Iacono M, Liuni S, et al. (2005) UTRdb and UTRsite: a collection of sequences and regulatory motifs of the untranslated regions of eukaryotic mRNAs. Nucl Acids Res 33: D141-146.
5. Bakheet T, Williams BRG, Khabar KSA (2006) ARED 3.0: the large and diverse AU-rich transcriptome. 34: D111-114.
6. Wu L, Wells D, Tay J, Mendis D, Abbott M, et al. (1998) CPEB-mediated cytoplasmic polyadenylation and the regulation of experience-dependent translation of α-CaMKII mRNA at synapses. Neuron 21: 1129-1139.
7. Matsuoka Y, Shibata S, Ban T, Toratani N, Shigekawa M, et al. (2002) A chromodomain-containing nuclear protein, MRG15 is expressed as a novel type of dendritic mRNA in neurons. Neurosci Res 42: 299-308.
8. Watt Terry J. DDF (2005) ESPSearch: a program for finding exact sequences and patterns in DNA,RNA or protein. BioTechniques 38: 109-115.
9. Miyashiro KY, Beckel-Mitchener A, Purk TP, Becker KG, Barret T, et al. (2003) RNA Cargoes Associating with FMRP Reveal Deficits in Cellular Functioning in Fmr1 Null Mice. Neuron 37: 417-431.

10. Darnell JC, Jensen KB, Jin P, Brown V, Warren ST, et al. (2001) Fragile X Mental Retardation Protein Targets G Quartet mRNAs Important for Neuronal Function. Cell 107: 489-499.
11. Gerber AP, Luschnig S, Krasnow MA, Brown PO, Herschlag D (2006) Genome-wide identification of mRNAs associated with the translational regulator PUMILIO in Drosophila melanogaster 10.1073/pnas.0509260103. PNAS 103: 4487-4492.
