# Supplementary material for: Transcriptome Analysis of Synaptoneurosomes Identifies Neuroplasticity Genes Overexpressed in Incipient Alzheimer's Disease
Source: PLoS One. 2009 Mar 19;4(3):e4936. doi: 10.1371/journal.pone.0004936 (PMC2654156; doi:10.1371/journal.pone.0004936)
Supplement: Table S1 — Neuropsychological and Neuropathological Assessment of Patients. The IAD group and the normal control group were not statistically different with regard to age, years of education, gender distribution, or interval between testing and death. As expected, the IAD group obtained lower scores on the MMSE than normal controls (p<0.01). MMSE scores ranged from 25 to 29 for normal controls and from 21 to 27 for IAD. No statistically significant difference was detected between NC and IAD for performance on either the composite measure or Digit Span backward. Statistical comparisons on each measure were made by Mann-Whitney U tests. (0.02 MB PDF) [file pone.0004936.s002.pdf]

Table S1: Patient Data

Neuropathological and Neuropsychological Assessment of Patients

								Executive Function		Memory	
	sex	age	PMI	MMSE	CDR	Amy	NFT	Composite	DS bkwds	Recall	Learning
Control	m	88	2	29	0	0	0	**7**	**1.8**	**-0.11**	**-1.11**
	m	88	4	29	0	0	0	**7**	**0.86**	**-0.67**	**-0.57**
	m	88	5	29	0	0	0	**7**	**1.8**	**1**	**1.6**
	f	91	4.5	28	0	0	1	**6**	**na**	**0**	**-1.7**
	f	99	9	28	0	1	1	**6**	**-1**	**-1.70**	**-2.9**
	m	83	3	28	0	1	0	**7**	**2.67**	**1.56**	**0.78**
	f	84	6	27	0	0	0	**7**	**-0.24**	**-1.60**	**-2.3**
	f	90	5.75	25	0.5	0	0	**7**	**NA**	**-2.5**	**0**
IAD	m	96	5	25*	1	0	0	**7**	**-0.1**	**-1.60**	**-2.3**
	m	85	4	26	0.5	0	1	**NA**	**-0.1**	**-2.30**	**-1.9**
	m	85	4.5	26	1	1	1	**6**	**-2**	**0.00**	**-2.3**
	f	95	5.75	25	1	1	0	**4**	**-1.05**	**-2.5**	**-2.7**
	f	94	4.5	22	0.5	0	0	**7**	**0.86**	**-2.89**	**-1.92**
	f	89	8	21	0.5	1	1	**7**	**-0.1**	**-2.30**	**-3.8**

Statistical Evaluation of Patient Data

	NC (n = 8)		IAD (n = 6)	
	M	(SD)	M	(SD)
**Gender (male/female)**	**4/4**		**3/3**	
**Age**	**88.4**	**(4.71)**	**91.3**	**(7.00)**
**Education**	**15.1**	**(2.41)**	**17.4**	**(2.41)**
**MMSE**	**27.9**	**(1.36)**	**24.5**	**(2.43)**
**Testing to death interval (months)**	**11.8**	**(9.96)**	**8.0**	**(6.55)**

Statistical Evaluation of Executive Function Assessment

	NC		IAD		p-value
	N	Median	N	Median	
**Composite**	**8**	**7.00**	**4**	**7.00**	**.62**
**Z_DS Backward**	**6**	**1.33**	**6**	**-0.10**	**.13**
