# Supplementary figures and images for: Transcriptome Analysis of Synaptoneurosomes Identifies Neuroplasticity Genes Overexpressed in Incipient Alzheimer's Disease
Source: PLoS One. 2009 Mar 19;4(3):e4936. doi: 10.1371/journal.pone.0004936 (PMC2654156; doi:10.1371/journal.pone.0004936)

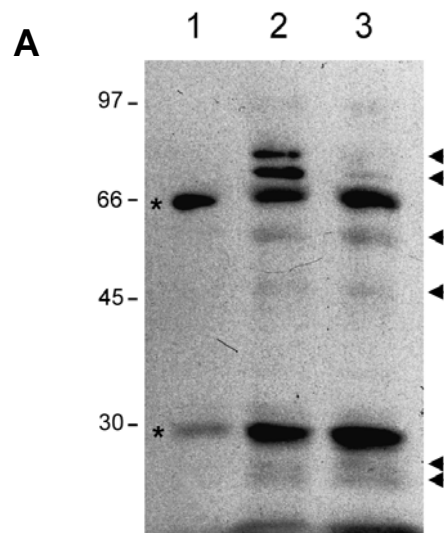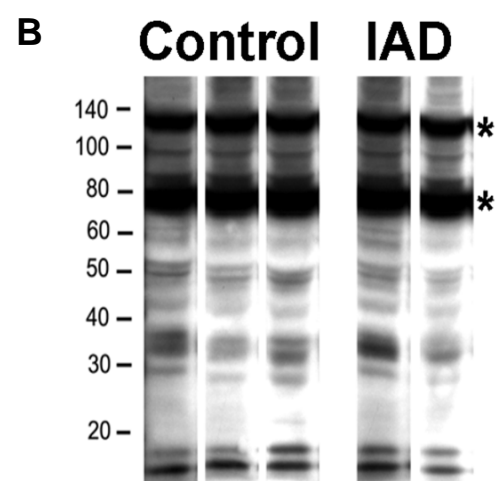

Supplement: Figure S1 — Functional Stability of Synaptoneurosome mRNA. A)To test stability of mRNA from synaptoneurosomes, pooled mRNAs from control tissues were combined with rabbit reticulocyte lysates (RRL) and Transcend™ tRNA, which is an ε-labeled, biotinylated lysine-tRNA complex with a detection sensitivity of 0.5–5 ng of protein, and the resulting biotinylated proteins detected by Western immunoblot. In combination with rabbit reticulocyte lysate (RRL) and Transcend™ biotinylated t-RNA the isolated mRNAs from control (lane 2) and AD tissues (lane 3) yielded several biotinylated species indicative of newly synthesized proteins seen between 70–90 kD (two bands), ∼60 kD and ∼50 kD, and two bands >30 kD. The identity of these proteins is undetermined. A no-template control, with RRLs only, shows that bands at ∼70D (also in Control and AD lanes) and 30 kD are endogenously biotinylated proteins (asterisks). B) In vitro translation function of synaptoneurosomes is maintained in the 5 patients tested, regardless of clinical stage of disease. Asterisks denote endogenously biotinylated species. (0.20 MB PDF) [file pone.0004936.s007.pdf]

**A**

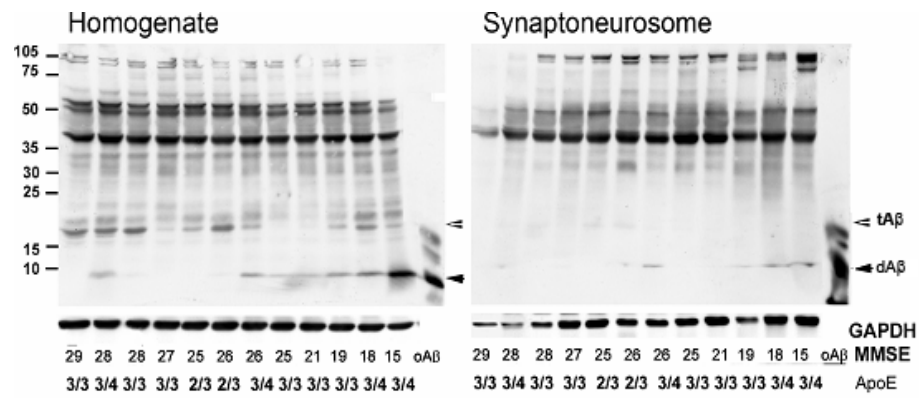

**B**

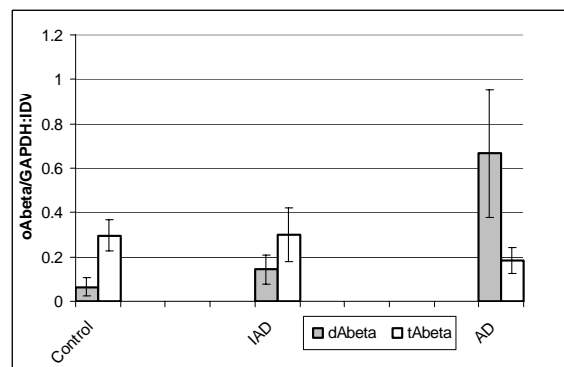

**C**

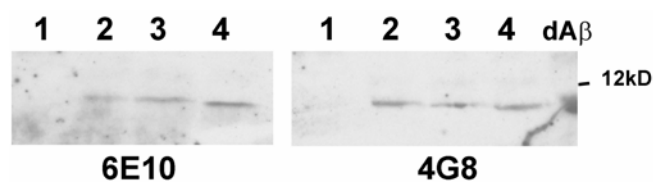

Supplement: Figure S2 — Immunoblots of Homogenates and Synaptoneurosomes Probed with MAbs 4G8 and 6E10. A) Only the band coinciding with dimeric Aβ (∼9 KD, arrowhead)is correlated with declining MMSEs or ApoE genotype in homogenates or synaptoneurosomes. B) Densitometry reveals that, when normalized to GAPDH, dimeric Aβ but not tetrameric Aβ levels are inversely related to MMSE. Because of small n and variability within groups, differences between groups is not significant . dAβ C v IAD p = 0.35, C v AD p = 0.12. tAβ C v IAD p = 0.99, C v AD p = 0.44 C) Immunoblots of control (lane1), IAD (lane2) and AD patients (lane 3,4) probed with antibodies 6E10 and 4G8 revealed labeling of dAβ only in patients with cognitive decline. (0.10 MB PDF) [file pone.0004936.s008.pdf]

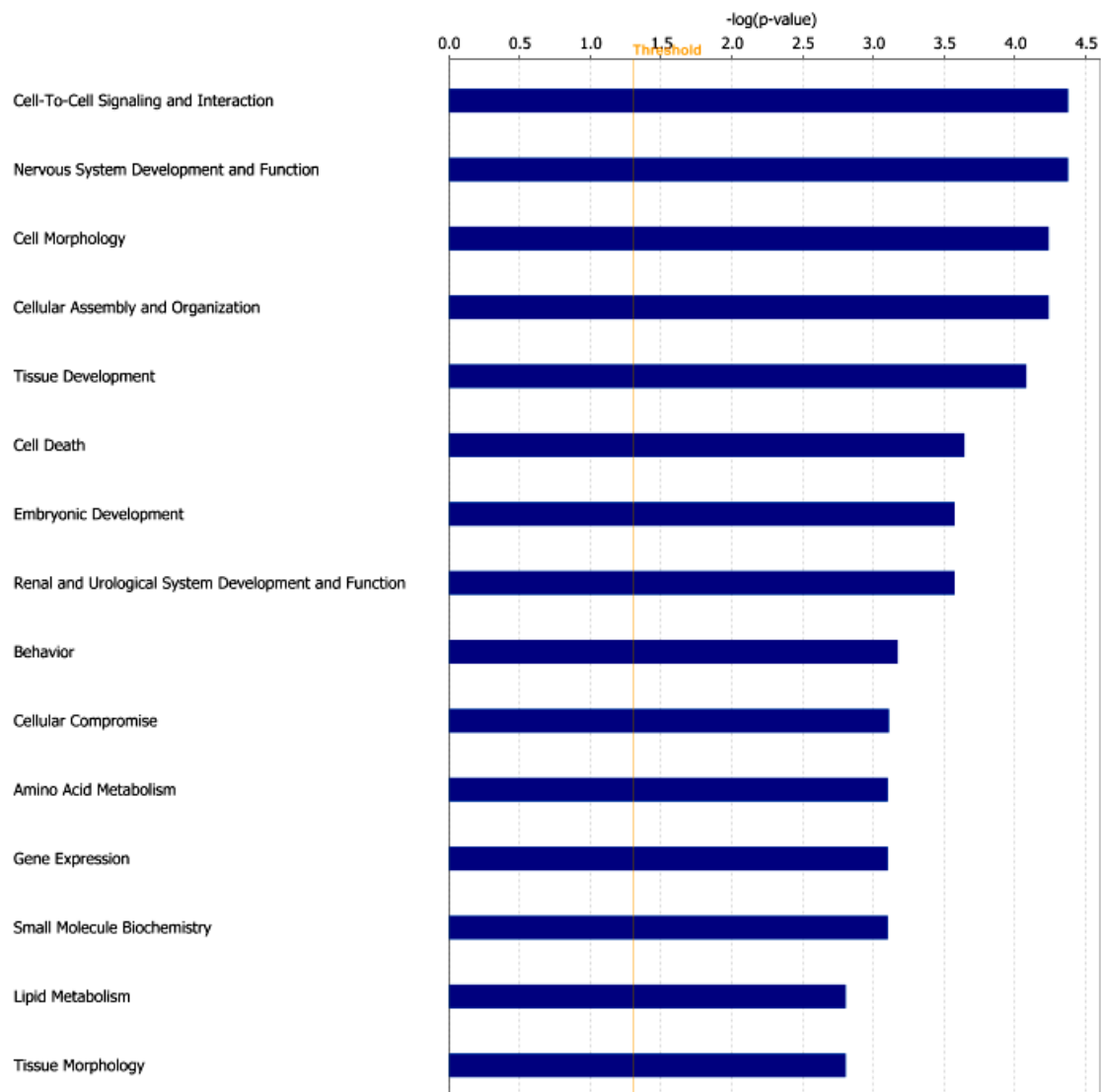

Supplement: Figure S3 — Ingenuity Functional Analysis. The Functional Analysis identifies the biological functions and/or diseases that were most significant to the data set. Genes from the p<0.01 dataset with fold change ≥1.2 in IAD patients were used for the analysis. Fischer's exact test was used to calculate a p-value determining the probability that each biological function assigned to that data set is due to chance alone. Threshold is at 1.3 = −log (p<0.05). (0.03 MB PDF) [file pone.0004936.s009.pdf]
