# Supplementary material for: Transcriptome Analysis of Synaptoneurosomes Identifies Neuroplasticity Genes Overexpressed in Incipient Alzheimer's Disease
Source: PLoS One. 2009 Mar 19;4(3):e4936. doi: 10.1371/journal.pone.0004936 (PMC2654156; doi:10.1371/journal.pone.0004936)
Supplement: Figure S4 — Network representation of the biological processes generated by genes upregulated in IAD. The Neurological Disease Network contains 37genes, 22 of which are focus genes upregulated in IAD (Figure S4A). Increased expression of two mRNAs encoding gamma-aminobutyric acid (GABA-A) receptors (GABRA1 and GABRA2) and one GABA-B receptor (GABBR2) in IAD patients, may indicate GABAergic terminal sprouting and suggests that inhibitory compensatory mechanisms may also be activated The Nervous System Development and Function Network (Figure S4B) contains 26 genes 17 of which are upregulated in IAD. In Figure S4C, Network 4 in subcellular layout, the functional significance of GluR2 (GRIA2) is seen by the addition of up- and downstream interacting molecules to the network. Notably, GluR2 is a potential target of FMRP. Other significant GluR2-interacting molecules, dynamin, AP-2 and Homer, are not represented in this chart. Genes overexpressed in IAD are seen in pink with fold change. All networks are listed in Table S4. (0.71 MB PDF) [file pone.0004936.s010.pdf]

Network 3 : Neurological Disease

A

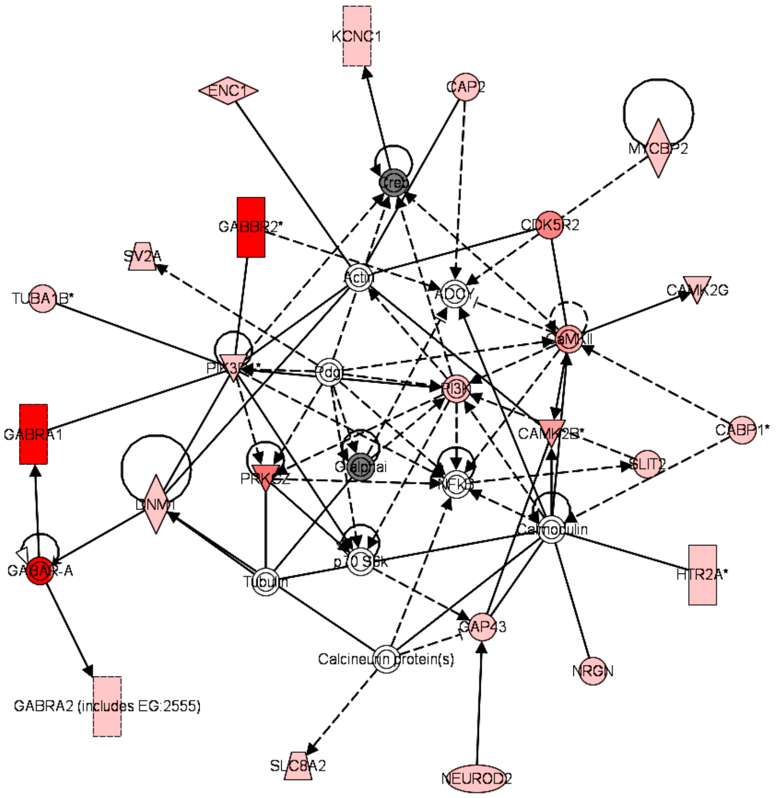

© 2000-2008 Ingenuity Systems, Inc. All rights reserved.

Key

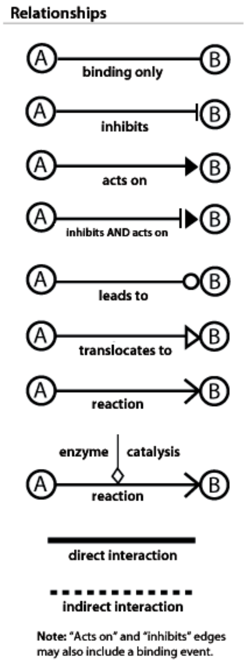

B

Network 4 : Nervous System development and Function

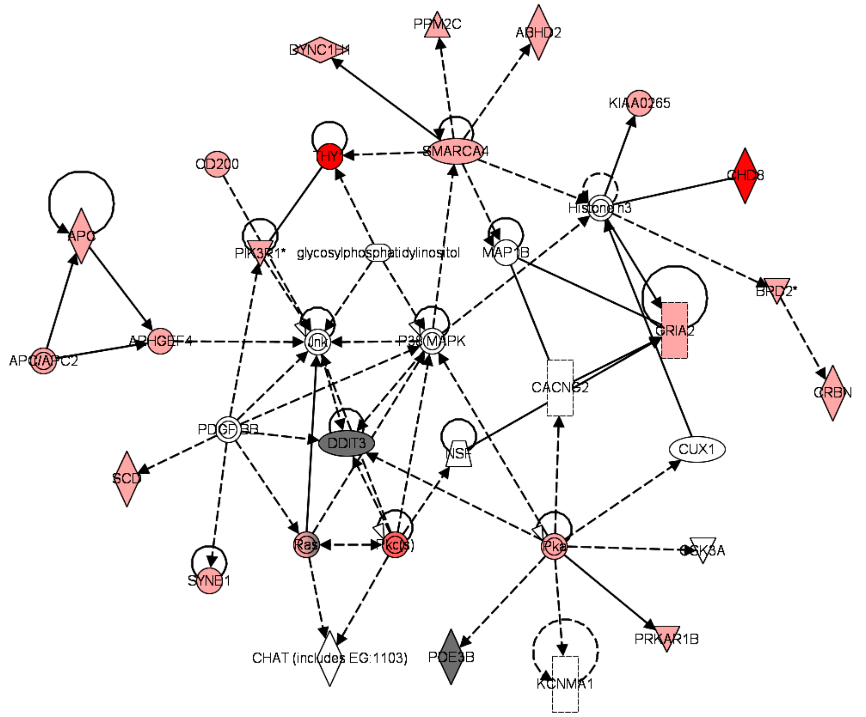

© 2000-2008 Ingenuity Systems, Inc. All rights reserved.

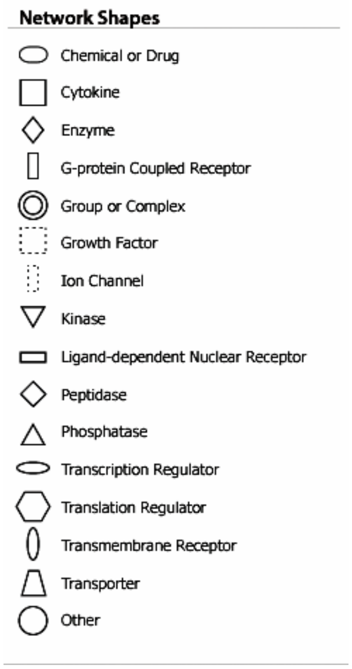

C

Network 4 : **GluR2 and Interactors**

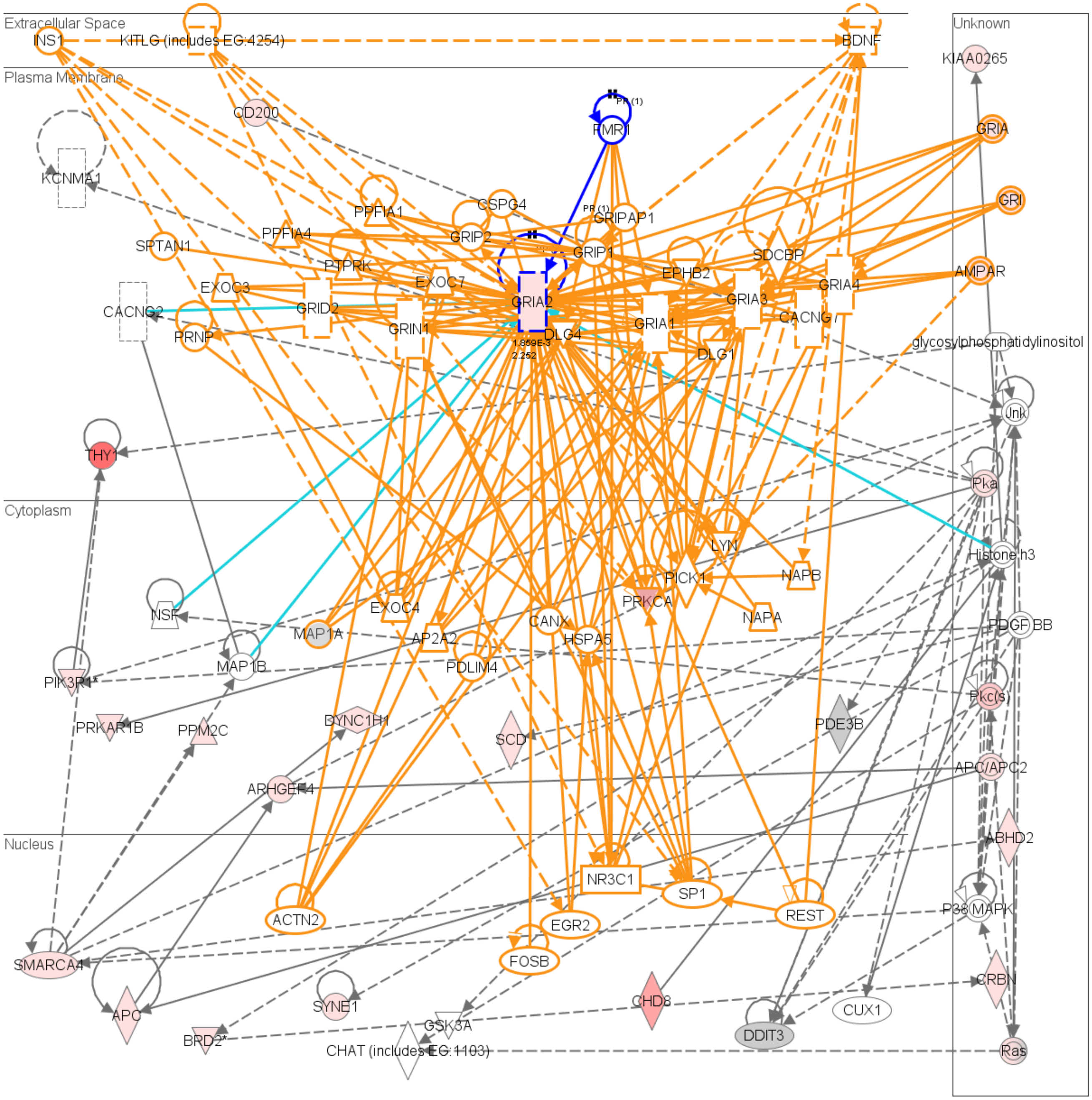

© 2000-2008 Ingenuity Systems, Inc. All rights reserved.
